# “Like Throwing a Bowling Ball at a Battle Ship” Audience Responses to Australian News Stories about Alcohol Pricing and Promotion Policies: A Qualitative Focus Group Study

**DOI:** 10.1371/journal.pone.0065261

**Published:** 2013-06-05

**Authors:** Andrea S. Fogarty, Simon Chapman

**Affiliations:** Sydney School of Public Health, The University of Sydney, Sydney, Australia; Vanderbilt University, United States of America

## Abstract

**Introduction:**

Policies affecting alcohol’s price and promotion are effective measures to reduce harms. Yet policies targeting populations are unpopular with the public, whose views can be influenced by news framings of policy narratives. In Australia, alcohol taxation receives high news coverage, while advertising restrictions have not until recently, and narratives are highly contested for each. However, research specifically examining how audiences respond to such news stories is scant. We sought to explore audience understanding of news reports about two alcohol policy proposals.

**Method:**

From June to August 2012, 46 participants were recruited for 8 focus groups in age-brackets of young people aged 18–25 years, parents of young people, and adults aged 25 or older. Groups were split by education. Participants were asked their prior knowledge of alcohol policies, before watching and discussing four news stories about alcohol taxation and advertising.

**Results:**

Participants were clear that alcohol poses problems, yet thought policy solutions were ineffective in a drinking culture they viewed as unamenable to change and unaffected by alcohol’s price or promotion. Without knowledge of its actual effect on consumption, they cited the 2008 alcopops tax as a policy failure, blaming cheaper substitution. Participants had low knowledge of advertising restrictions, yet were concerned about underage exposure. They offered conditional support for restrictions, while doubting its effectiveness. There was marked distrust of statistics and news actors in broadcasts, yet discussions matched previous research findings.

**Conclusions:**

News coverage has resulted in strong audience understanding of alcohol related problems but framed solutions have not always provided clear messages, despite audience support for policies. Future advocacy will need to continue recent moves to address the links between alcohol’s price and promotion with the drinking culture, as well as facilitate understandings of how this culture is amenable to change through the use of evidence-based policies.

## Introduction

Health and social costs associated with Australian alcohol consumption [1] underscore high priority for policy responses that reduce the prevalence of community harms [2]. In 2009, the evidence for a range of policy priorities were assessed and recommendations outlined [3]. Among these, two have the potential for population-wide reach: alcohol pricing and taxation, and restrictions on alcohol advertising.

Taxation aims to affect alcohol’s price [4], with higher taxation for high alcohol drinks and preferential tax for drinks with lower concentrations of alcohol. Alcohol concentration is not uniformly taxed in Australia, with drinks taxed by beverage class (beer, wine or spirits) and subject to different rates that favour higher alcohol percentage wine [5]. In 2008, the Australian government commissioned a review of Australia’s tax system, to improve economic efficiency for future years by recommended changes to the structure of taxation and transfers [6]. Known as Henry Tax Review, the 2010 report concluded that alcohol taxation should be re-evaluated, and recommended volumetric taxation, where the tax applied refers to the amount of alcohol in a drink, rather than beverage class [7]. This could be a flat volumetric tax [8], or public health’s preferred solution: a tiered tax with increasing tax ‘bands’ tied to increasing percentage alcohol in drinks [5]. An existing pricing policy based on beverage class, is the 2008 ‘alcopops tax’ which increased the tax applied to ready-to-drink (RTD) spirit mixes [9]. Other potential approaches include a minimum ‘floor price’ for a standard drink, below which alcohol cannot be sold [10].

Restrictions on alcohol advertising in Australia rely upon an industry voluntary agreement, the Alcoholic Beverages Advertising Code (ABAC) [11]. In turn, the code and television advertisements are subject to the Commercial Television Industry Code of Practice (CTICP) [12] and the Australian Association of National Advertisers Code of Ethics [13]. These include restrictions on the timing and placement of advertising (e.g. no television advertising before 8.30pm unless during live sports broadcasts) and on their content (e.g. not depicting underage people). However, such codes fail to prevent underage exposure [14] and existing guidelines are breached [15]–[18]. Complaints are made to the Advertising Standard Board (ASB) [19] yet are rarely upheld, prompting the formation of the Alcohol Advertising Review Board (AARB) by public health organisations. The AARB aims to provide an independent avenue for complaint [20].

Acceptance of alcohol policies depends on community attitudes towards harms and understanding of policy solutions. A national poll found 80 per cent of all Australians think there is problem with excessive alcohol consumption. Support increased with age, and over a third perceive alcohol to be the most harmful drug [21]. Young people and their parents are highly aware of the negative impacts of alcohol, particularly with regard to mental health [22]. However, attitudes towards policies are mixed. Older Australians, non-drinkers and those with teenagers are more likely to support tax policies than other groups [21]. The more alcohol people consume, the greater the opposition to taxation [23]. Police and the public are more supportive than licensees regarding licensed premises strategies [24]. Punishment of drunk patrons, and harsher penalties for drink-driving, are more popular than reducing alcohol’s accessibility [25]. A review found that targeted policies (e.g. penalties for irresponsible service of alcohol) are more palatable than universal policies (e.g. taxation) [26]. However, with alcohol advertising, support for greater regulation of promotions by an independent body is high [21], [26], especially regarding sport [27].

A key element influencing community perceptions is how news stories frame policy narratives. A large body of research has investigated the power of news media to shape dominant definitions of what is at issue and solutions deemed appropriate [28]–[30]. As Entman wrote, to frame is to “select some aspects of a perceived reality and make them more salient… in such a way as to promote a particular problem definition, causal interpretation, moral evaluation and/or treatment recommendation” (p52 [30]). This is explicitly recognised by advocates who seek to shape audience interpretations of alcohol news stories [31], [32]. News coverage of alcohol control is highly contested, where public health and drinks industry news actors promote differing policy solutions, based on different organisational aims [33]–[36].

In 2009, alcohol and substance use ranked eighth among health stories on Australian television news [37]. Regular attention to alcohol represents a significant opportunity for policy advocates to continue advancing key messages and for researchers to explore the dominant messages being conveyed. An analysis of all Sydney television news stories about alcohol between 2005 and 2010 [38] shows that news paints a substantial picture of the ‘problem’ of alcohol and attendant health effects, but that reportage of alcohol-control policies was scant and unaligned with identified priorities, with the notable exception of the ‘alcopops tax’ [9].

In newspapers, alcohol advertising restrictions have historically experienced low newsworthiness and are contested by a range of public health and industry voices [39]. In recent years, groups like the AARB and the National Alliance for Action on Alcohol (NAAA) [40] have made media advocacy concerning alcohol advertising a top priority [41], likely affecting the news coverage. Likewise, newspaper coverage of the alcopops tax [9] saw public health representatives stressing potential health benefits, while opponents stressed potential substitution effects and accused the government of a ‘tax grab’.

While the aforementioned national poll gives insight into the public’s support for alcohol control policies, and news content analyses reveal the narratives in play in the news, there is little research examining how these factors relate to each other in the audience. This is important for refining future key messages and advocacy efforts concerning alcohol. In this paper, we examine audience understandings of news broadcasts about alcohol taxation and advertising policies. We hypothesised (i) audiences would agree there was an alcohol ‘problem’ in Australia, (ii) attitudes towards the alcopops tax would reflect dominant arguments seen in newspaper coverage, and (iii) audiences would have limited understanding of alcohol advertising restrictions due to its past low newsworthiness and standing as a ‘proposal’.

## Methods

### Ethics Statement

The study protocol was approved by the University’s Human Research Ethics Committee.

### Focus Groups

We used semi-structured focus group discussions to examine audience responses. Participants were recruited via email networks, study information flyers, and ‘snowball’ referrals. Using groups identified in prior research [21], we aimed to recruit drinkers and non-drinkers in the following age brackets: 18–25 years; 25+ years; and parents of young people (i.e. aged under 25). It proved difficult to recruit non-drinkers. We ran mixed sex groups within these age ranges, split by level of education. Participants were compensated $A50 for their time.

### Data Collection

Between June and August 2012, eight focus groups (n = 46) were facilitated by author AF and each ran for about one hour (Table 1). Participants were given information about the study and opportunity to ask questions before signing written consent forms. Discussion was encouraged, with assurances there were no wrong answers. Discussions were recorded and transcribed for analysis. Participants were asked about their news habits, recollections about alcohol news stories and knowledge of policies before prompted discussions about taxation and advertising. Participants were shown four news clips concerning (i) the 2008 alcopops tax, (ii) conflicting data about the impact of the alcopops tax, (iii) proposals to restrict alcohol advertising in professional sport and, (iv) reports that broad advertising restrictions could result in lower consumption and lower morbidity. Display order was alternated, so that half discussed advertising first, while others started with the alcopops tax.

**Table 1:** Characteristics of focus groups recruited.

Group	n	Agegroup	Education	Individual gender and age
1	8	25+	Mixed	F34, F33, F29, F27, F35, F33, F32, F33
2	6	18–25	Secondary/tertiary	F24, M18, M18, M19, F18, M25
3	7	Parents	No tertiary	F?, F51, F31, F41, F35, F54, M58
4	6	25+	Secondary	M?, F38, M33, M32, F25
5	5	Parents	Tertiary	M45, F25, F46, M33, M54,
6	4	25+	Tertiary	F55, M50, M39, F44
7	6	18–25	Undergraduates	F21, M23, F20, F19, F24, M45
8	4	18–25	Undergraduates	F23, M24, F19, F20

### Data Analysis

Transcripts were analysed using NVIVO 10. The interview schedule, a previous interview study [42] and news content analyses [9], [38], [39] were used to develop initial codes and we allowed further codes that arose within the data. Coding was trialled and refined on two transcripts, before being applied across the remainder. The two trial transcripts were then recoded at the end and included in the analysis.

## Results

### Alcohol in the News

Participants reported active news-seeking and passive exposure to news. With the exception of the parents’ group, television news was actively rejected as low quality. Many described preferred news sources, such as individual RSS feeds.

A clear characterisation of alcohol stories arose in all groups: *“That it's bad. That it's infecting society. That it's corrupting all the youths and everyone's going crazy”* (F19). Recollections of commonly arising narratives related to youth, drunkenness and violence: *“I just remember like the footage on telly of really trashy girls in the street, just like falling over each other…”* (F28).

Younger participants reported an inability to identify with news portrayals of young people. They perceived that the news made unwarranted generalisations and some actively rejected news stories as a result of their own experiences: *“…they keep talking about people getting beaten up on the street…but when I go out I hardly ever see it…”* (M23). Several parents thought the dominant focus on violence and accidents resulted in omissions of important information, such as longer term effects on health. They felt the emphasis on binge drinking allowed young people to ignore the risks posed to their health in later years.

### Understanding of Alcohol Policies

Participants reported limited policy knowledge beyond state-based venue licensing and policing of alcohol related violence. Most related policies to antisocial behaviour instead of measures aimed at the wider population. While some mentioned pricing measures, only one detailed specifics: *“…the government enforces a regulation whereby alcohol venders have to charge a minimum, for certain or all, products I assume, I don't really know…”* (M24).

A common pattern emerged where, unprompted, many reported misgivings about the effectiveness of policies, as alcohol-related problems were seen as both too complex and too socially embedded for any policy to create meaningful changes. They felt that suggested policies were over-simplified and too targeted to address a problem they saw as huge and potentially intractable: *“it’s like throwing a bowling ball at a battle ship…” (M33).*

One person perceived confusion in public discussion about the aims and reach of specific polices and thought that this information would be useful for the public to have in order to understand the issues: *“…none of them have stated what the desired end goal is. Is it a reduction in road fatalities? Is it a reduction in liver cancer? … Even the politicians who are proposing for and against them, haven't really spelt out what it is they're trying to achieve. They're just giving us a vibe this would be a good idea.*” (M33).

Younger participants perceived themselves as commonly the target of such measures, yet thought the problem existed more largely in society, pointing out that older people consumed alcohol and such consumption was seen as socially acceptable, not problematic. They felt that focusing on young people was hypocritical and asserted that those voicing policies also drank when they were young, so questioned what had changed.

### Alcohol Advertising Restrictions

Participants had low knowledge regarding advertising restrictions, with little understanding of whether these were legislated, what form they might take and who was responsible for oversight. Most commonly, they reported restrictions on television advertisements only and were unsure of the curfew’s time: *“I know they’re not allowed to advertise before a certain time in the day. They’re not allowed to advertise during children’s programming. I’m not sure of any other limitations…”* (M32). Only one mentioned that restrictions were voluntary and expressed cynicism that they were adhered to. The same person mentioned restrictions on content of advertisements, yet acknowledged they had never known the details. Nobody mentioned the ABAC guidelines by name or the Advertising Standards Board.

Some denied advertising affected consumers while others thought effects were self-evident: *“…that's the nature of the beast isn't it? Really, I mean if it didn't work they wouldn't do it*” (M45). Some thought advertising effects receded with age: “*I guess when you get older, maybe you tune out to the ads, it's not important”* (M45), while others thought other factors, such as parenting, could overcome advertising effects. These discussions were debated within groups, with some reconciling the differences to brand preference: “*I think the advertising is more to get people to decide what to drink rather than to start drinking or not drinking”* (F24).

The news clip discussing sport advertising restrictions featured a politician and three sporting administrators, causing some to reject it out of hand: “*I just roll my eyes when I hear his name now so I’m usually not looking at the screen while he’s talking*” (M32). Some noted the absence of health news actors, who they felt would have leant credibility to the news broadcasts. In particular, parents thought featuring sport-CEOs obscured health issues, stating that CEOs had mostly economic concerns and did not care about a health angle. Parents were concerned about the impact these sorts of actions had on people’s lives in the community: *“There's a self-interest, at the cost of maybe community values and how it's going to impact everyman's life…”* (F41) More than other groups, parents saw mutually vested interests in relationships between sporting and alcohol companies: “…*the organisers want the advertising because of the money but the advertisers must want to advertise, otherwise if it didn't work for them they wouldn't do it…*” (F51). This concept of was echoed occasionally in other groups, where some thought CEO news actors were interested in preserving salary bonuses and questioned why sport was allowed to have alcohol advertising, when the 830 pm curfew acknowledges that a problem exists.

However, some could not see advertising restrictions being effective: “I think that they're going to drink beer watching sport regardless if there are ads or not. So I don't see why it would make a difference” (F18). Younger participants were more likely to view alcohol advertising in sport as unproblematic.

Despite conflict about the effectiveness of restrictions, participants were united regarding advertising to children: “I'm a bit torn because I do really like sport. I am aware that a lot of high level sport does survive on corporate partnerships. But I'm really feeling that I don't see why it has to be alcohol and I do think that a lot of young kids [are] watching live sport…” (M33). Two participants consistently objected to this though: “I mean yes we should be protecting kids…but it's adult drinking that's a problem. You're thinking of these kids when they grow up and start drinking, so what about the adults?” (F23).

All participants expressed some conditional support for advertising restrictions, with most arguing the code of practice should be followed by advertisers, monitored by the government and have stronger punishments for breaches. Parents gave unqualified support, more than in other groups, with their children in mind: “*Having kids I know that they watch the TV. They’re very impressionable.* (F?).

### The ‘alcopops’ Tax

Participants reported hazy recollections of the 2008 tax: “I do remember hearing it. Pretty much all I remember is they wanted to bring up the price of the alcohol sort of more appealing to the younger people… in some sort of effort to curb the amount they’re going to drink, which is never going to happen” (M32). Some were clear that it was failed exercise: “The tax thing that didn't work” (F19), while others were less sure: “We don’t know if it worked. We have got no idea” (M34).

None related personally to the tax, describing it as something happening to other people, citing their current age as a factor in ignoring the news on alcopops. Even those who were in the target age range at the time positioned RTDs as drinks that other people drank. Others described it as purely a matter of politics: “*…each party bickering at each other like they usually do about every issue… So it was just a lot of blah, blah, blah from either side of the house and not really anything constructive”* (M50).

Discussion of the two alcopops news stories resulted in recognisable themes, as identified elsewhere [9]. The most common response, by far, was that consumption would be unaffected and drinkers would just switch drinks by substituting something cheaper. This was expressed by parents and young people alike, with some even relating it recent experiences while drinking: *“exactly the decision we made the other weekend, it was whether to buy pre-mixed vodka or normal vodka. We went with normal vodka because it was cheaper”* (M32).

Even facing two sets of opposing data about the tax’s effect, where different news actors argued for different effects, people stuck to their position: *“I think everyone here agrees that people will just find something else to drink if they can’t afford the alcopops”* (M50). Of the eight groups, only two initially recognised that there were two distinct sets of data presented in the clip which concluded opposite effects. The remaining groups reported they did not understand the figures at all, or that the figures proved substitution occurred. Even when prompted by the facilitator to discuss the different results, they often negotiated a position that maintained substitution was the result: *“I don't know, it's tough… there would have been at least a single individual in the country who drank less as a result… But I mean once you've already started down that path of wanting to get drunk and very drunk often, it wouldn't really stop you.”* (M24).

A second theme emerged, where a few displayed cynicism about the tax’s presumed concern for health, instead viewing it as a revenue-raising tax grab. Some characterised it as a sinister move by the government to receive money, without intending to solve any problems: *“All I see is somebody’s cashing in somewhere”* (M34). Participants engaged in hypothetical discussion of how they thought consumption reduction might have occurred, including anecdotal evidence from personal experiences: *“I don't know I guess it is sort of true. I mean even nowadays you'd never ever see anyone drinking alcopops… maybe I'm just getting older*” (M23). Others considered possible mechanisms for change, guessing that even with substitution, some people would still not be able to afford buying a bottle of spirits and would continue to buy alcopops, even if it meant buying less of them after the tax. One argued that drinks industry objections meant the tax had been effective, reasoning that if the tax wasn’t working they would be making more money from sales and so wouldn’t complain about the tax. Some conceded it might be part of package of solutions but doubted it would have a big impact on its own.

For those with little recall of the tax, seeing news clips did not clear up any confusion. Many thought it was difficult to know what to think and felt the news had not communicated information clearly enough: *They were saying there was a shift to increased purchasing of spirits, but that was kind of refuted. So you don't really know unless you look at the figures yourself”* (F46). One saw this as intrinsic to news production: *“It’s designed to confuse you.”* (M32), while another thought it represented vested interests :: *“you’ve got two parties that are both going to give you wrong data for whatever reasons to make themselves look good or to make a profit. So, I mean, it’s really, really questionable; the two sources in the news*.” (M45).

### Mistrust Data

Across all clips, a pattern emerged of mistrusting research data presented in the broadcast, regardless of topic or news actor. Nothing was taken at face value, unless confirming their position. For example, when discussing advertising bans, many could not fathom the presented link between restrictions and subsequent reductions in morbidity: *“I thought those figures sounded like it was a bit too good to be true. Like a 25 per cent reduction in, I think, alcohol consumption and a 30 per cent reduction in the road toll or something. The figures seemed a bit too high. I don't know if I'd believe that…”* (F28). When asked about their disbelief, participants cited not knowing enough about the research being reported, a disconnection with figures in general, and a lack of specificity: *“There's always figures within figures too. It's 25 per cent of what? Is it 25 per cent of something that happened last month or is it over a year or…”* (M45) *“And is it everyone or is it just young people or…*” (F46).

The changing nature of data was invoked discussing alcopops tax figures about consumption reduction: *“It could be, well you might reduce it for a short period of time. But then in a year or two things might change.”* (M51). This sentiment expressed in the parents’ group was also supported by younger people who suggested that data could be found by any interested party to fit their ideas. Even in the final advertising clip, where only one set of uncontested figures was reported, they remained sceptical: *“Yes, they just said research shows this and if you've lived in this society for even a small amount of time you know that a couple of years later research can just say something else”*(M24).

### Drinking Culture

Though not directly asked, all groups keenly highlighted the ‘drinking culture’, which they viewed as more important to consumption than alcohol’s price or promotion. When pressed, their ability to articulate what the drinking culture was, or how it arose, was limited yet they expressed functioning within it. Parents expressed concern for children facing peer pressure in the current drinking culture, while others thought it was just part of our natural history: “*…it's been part of our culture since we’ve been standing on two feet basically. So it’s really hard to sort of take a step away from that*” (M32).

They could not clearly describe how it affected their drinking choices, but nevertheless, asserted that it did and, on the whole, denied advertising and price helped create or maintain the culture. Only one group openly considered this. Instead, it was viewed as the biggest obstacle to progress in reducing alcohol-related harms.

## Discussion

Our results provide fresh insight into audience responses to news stories about two alcohol policies and to what extent dominant news frames already identified in news coverage were present in audience understandings. We found that while participants agreed alcohol consumption in Australia causes significant social problems, this was tempered by scepticism that current policy options have the capacity to reduce these problems. This reflected, in part, misunderstandings of the extent to which policies are expected to work, as well as a lack of familiarity with the arguments and evidence for particular proposals. Participants also keenly emphasised the role of the ‘drinking culture’, which in Australia can involve increases in assault and hospitalisations around public holidays and cultural events [43]. Yet participants did not view current lack of alcohol regulations as connected in some way to the problematic culture they described. They viewed the place of alcohol in Australian culture as so deeply entrenched as to present a formidable and perhaps intractable barrier to the success of any policy.

Policy advocates can be heartened that dominant messages about alcohol-related harm identified in news media [38] are generally reflected in audience discussions, where people readily report understanding the ‘alcohol problem’, yet are less clear about the role for policy solutions. This represents a key area for policy advocacy: to continue current approaches to advocacy which seek to move from defining the alcohol problem towards extended foci not just on solutions, but also explaining precisely how and to what extent these solutions work synergistically. Participants in this study reported limited understanding of alcohol policies beyond measures in place at licensed premises concerning alcohol sale. They rejected outright news narratives that pricing and promotions policies could provide viable solutions, reasoning they personally would not be affected by such factors, thus failing to see how policies connected to the problems they had earlier agreed existed. The challenge then concerns shaping dominant news narratives in such a way that audience members explicitly understand the evidence-based links which implicitly underlie alcohol experts’ policy advocacy in news media.

It is also clear that our audience members did not conceive of policies as set of options that work in concert, and instead thought of them individually, perceiving them doomed to failure. For example, our participants persistently understood the alcopops tax to have failed, despite existing data showing that the alcopops tax indeed produced overall net reductions in population consumption of alcohol [9], [44]–[47]. For some this reflected disbelief that the tax could work in the first place. More importantly, for others this arose from their recall of the public discourse in the tax’s aftermath. For our participants, drinks industry framings identified in public discourse about ‘substitution’ were endorsed more often than public health framings that asserted taxation affects consumption in predictable ways [9]. The substitution narrative was the most persistently expressed, regardless of age. In their experiences of the drinking culture, substitution more easily made sense than the more nebulous idea that it might work on a population-wide basis with a small net effect on consumption. As the tax is now long in place, correcting this misinformation is unlikely to be given any priority but it nevertheless provides useful information. Future policy advocacy, particularly around a volumetric tax, will need to reinforce not only that previous taxes have some measurable benefit, but also how exactly, this works at a community level rather than an individual one.

We note that since these discussions were conducted, the AARB has contributed to increased visibility of the issue of alcohol advertising and the potential for restrictions to help reduce problems. There has been attendant media coverage of both public health’s policy position and the alcohol industry’s response to the AARB [48]–[50]. While the effect of these activities on the public’s attitude has yet to be assessed, our results imply positive opportunities for future advocacy for possible legislation of alcohol advertising restrictions. Here, participants talked about restricting alcohol advertising in sport and preventing underage children from being exposed in a very similar way to public health narratives already identified in news coverage about advertising restrictions [39]. Yet encouragingly for advocates, participants did not oppose advertising restrictions in similar ways to drink industry framings of the issue. For example, our groups did not state that the drinks industry was ‘already responsible enough’, nor did they perceive proposals to restrict advertising as signs of ‘nanny’ interfering needlessly in their lives [39]. Instead, they were more likely to object on the basis that they couldn’t see how advertising restrictions would actually be effective. This implies that arguments against advertising restrictions currently do not have the same traction among audience members as arguments for such restrictions and there is role here for advocates in increasing knowledge about how advertising restrictions could affect consumption and morbidity rates.

To capitalise on the existing situation, advocates might note the following: knowledge of even the voluntary restrictions that exist was very low. No person we spoke with had heard of the ABAC scheme, nor did they know where complaints could be directed to regarding inappropriate advertising. This clearly points to the ongoing need for organisations such as the AARB to continue raising awareness of the issue. There was negligible understanding of the guidelines, with most only being familiar with a television curfew. This may reflect existing news coverage, where advertising was mostly referred to as ‘on television’ and stories focused on removing the curfew exemption for live sports broadcasts [39]. This suggests clear opportunities to improve knowledge of what exactly constitutes advertising and the standards to which it is expected to be held. This could tap into existing community concerns in a useful way: most groups conditionally supported advertising restrictions in principle, with the highest support reserved for those that focused on reducing underage exposure to advertising. Future advocacy can be encouraged that gaining the public’s support for restrictions will be arguably more simple than gaining their support for taxation policies.

However, coupled with these findings was the clear notion from many participants that advertising simply did not affect them, or merely focused on changing brand preferences and purchasing decisions. We suggest then, that capitalising on the community’s existing concern for children will need to focus on how, exactly, restrictions work to protect younger members of society. Key messaging could clarify that not only does advertising work to shape brand preference among drinkers, it can arguably increase consumption, as well as help normalise and glamorise alcohol for those too young to be regular consumers – those who participants are already concerned about.

Our results suggest significant possibilities for future advocacy concerning the notion of the ‘drinking culture’, which participants saw as overwhelming any potential benefits produced by alcohol policies. Their inability to articulate how the drinking culture arose and their denial that alcohol’s availability, price and promotion contributed to creating or maintaining a drinking culture, is a key area to target for public discussions. Raising greater awareness of the ways in which ‘culture’ is amenable to change seems crucial, when audience groups see the current drinking culture as historically inevitable. We suggest that future advocacy can better explicate other instances where cultural change has been possible (e.g. tobacco, drink driving), as well as the ways that interest groups attempt to influence the definition of what ‘normal drinking’ is and make the links between alcohol advertising, pricing and the ‘culture’ explicitly clear.

In our groups, it seemed to only be parents who clearly articulated concern about vested interests for the drinks industry and partnerships with sporting organisations, while younger participants were not as concerned. Like taxation, this may reflect a gap between their perceived individual experience of advertising having no effect on them and policies that focus on the population. Future advocacy concerning vested interests might encourage members of the audience to think less of their individual experiences and more about other, more vulnerable members of the community.

Lastly, advocacy should be aware of the lasting mistrust of statistics and figures presented in the broadcast. Our results show that audience members reported narratives from news broadcasts, not memories of statistics. Our suggestion then is that when presenting data, strong narratives about its significance and the impact of policies will be more important than quoting specific figures such as the expected percentage in mortality reduction.

### Conclusions

News coverage of alcohol stories has resulted in strong audience understanding of alcohol-related problems. However, the news framing of alcohol policy solutions has not always provided a clear cut-through message that audiences can understand, despite being supportive of policies as evidenced here. Future advocacy, particularly for taxation measures, will need to continue the recent moves that address the links between the drinking culture and factors such as alcohol’s promotion. For both alcohol’s price and promotion, future advocacy might help facilitate understandings of how this ‘drinking culture’ is amenable to change through the use of evidence-based policies.
